# Gastrointestinal Dysfunctions in Parkinson's Disease: Symptoms and Treatments

**DOI:** 10.1155/2016/6762528

**Published:** 2016-12-06

**Authors:** Andrée-Anne Poirier, Benoit Aubé, Mélissa Côté, Nicolas Morin, Thérèse Di Paolo, Denis Soulet

**Affiliations:** ^1^Axe Neurosciences, Centre de Recherche du CHU de Québec (Pavillon CHUL), Quebec City, QC, Canada; ^2^Faculty of Pharmacy, Laval University, Quebec City, QC, Canada; ^3^Department of Psychiatry and Neuroscience, Faculty of Medicine, Laval University, Quebec City, QC, Canada; ^4^Faculty of Medicine, Laval University, Quebec City, QC, Canada

## Abstract

A diagnosis of Parkinson's disease is classically established after the manifestation of motor symptoms such as rigidity, bradykinesia, and tremor. However, a growing body of evidence supports the hypothesis that nonmotor symptoms, especially gastrointestinal dysfunctions, could be considered as early biomarkers since they are ubiquitously found among confirmed patients and occur much earlier than their motor manifestations. According to Braak's hypothesis, the disease is postulated to originate in the intestine and then spread to the brain via the vagus nerve, a phenomenon that would involve other neuronal types than the well-established dopaminergic population. It has therefore been proposed that peripheral nondopaminergic impairments might precede the alteration of dopaminergic neurons in the central nervous system and, ultimately, the emergence of motor symptoms. Considering the growing interest in the gut-brain axis in Parkinson's disease, this review aims at providing a comprehensive picture of the multiple gastrointestinal features of the disease, along with the therapeutic approaches used to reduce their burden. Moreover, we highlight the importance of gastrointestinal symptoms with respect to the patients' responses towards medical treatments and discuss the various possible adverse interactions that can potentially occur, which are still poorly understood.

## 1. The Importance of Nonmotor Symptoms in Parkinson's Disease

In the early 19th century (1817), with the publication of* An Essay on the Shaking Palsy* [1], Dr. James Parkinson was the first to provide a clear clinical description of the disease that now bears his name [2, 3]. There are currently four motor features characterizing this neurological disorder, namely, muscle rigidity, tremor at rest, bradykinesia, and postural instability [3, 4]. However, a definitive diagnosis of Parkinson's disease (PD) is difficult to establish and can be obtained only* postmortem* by the demonstration of the presence of Lewy bodies [3]. Therefore, clinicians currently rely not only on motor symptoms manifestations but also on a positive response to levodopa (L-DOPA) treatment [4].

Progressive alterations of dopaminergic (DAergic) neurons in the nigrostriatal pathway are at the core of the abovementioned motor symptoms, resulting in a dysfunction of the somatomotor system. The extent of dopamine (DA) loss in the substantia nigra is already about 50–70% when the first motor symptoms emerge, and although PD is a progressive neurological disorder, DAergic deterioration is usually very slow and varies from one person to another [4]. An early diagnosis of the disease based on the Unified Parkinson's Disease Rating Scale (UPDRS) has a favorable long-term impact on the quality of life of patients [3].

Over the course of PD progression, motor impairments are generally preceded by nonmotor symptoms (NMS) such as depression, olfactory deficit, sleep behavior disorder, and constipation, sometimes by up to ten years [5–8]. In his essay, James Parkinson had mentioned some of these nonmotor features, namely, constipation, sleep disorders, dysphagia, drooling (sialorrhea), bladder dysfunction, and a slight state of confusion [1]. Nowadays, NMS are increasingly associated with PD, although they have not yet received extensive attention [6]. Indeed, patients report less than 40% of their nonmotor problems to healthcare professionals, either out of embarrassment or because these symptoms are seen as commonplace and inconsequential events [8]. To compound this problem, only a few NMS are recorded in medical files and are associated as such with PD, although those problems have been shown to result from the disease itself rather than being unremarkable manifestations of normal aging [9–12]. Therefore, these NMS, which are very often overlooked and are poorly investigated and treated, can have a major negative impact on the clinical care and quality of life of PD patients [6, 13–15]. Patients also often indicate that their NMS are more difficult to manage than their motor problems and may sometimes result in their hospitalization and institutionalization [6, 15, 16]. In addition, it has been demonstrated that attenuating NMS greatly improves the quality of life of patients, particularly those who positively respond to a DAergic therapy [15, 17]. Thus, the recently developed awareness on the detection of the different NMS early in the course of PD has led to a more critical appraisal of its etiology, the identification of risk factors, and the current advances in neuroprotective and therapeutic biomarkers of PD [5, 6, 18–20]. In light of these lines of evidence, PD can no longer be viewed solely as a complex disorder of motor functions, but rather as a progressive condition involving both motor and nonmotor features [5, 15, 21]. Some investigators have even proposed that PD could be divided into three phases, namely, preclinical, premotor (corresponding to the NMS), and motor phases [6, 20]. In some patients, nonmotor problems can be reminiscent of complications resulting from pharmacological and surgical interventions for the treatment of motor symptoms [16]. NMS can also be more predominant in the “off” medication state and some might be alleviated by DAergic therapy or, on the contrary, be exacerbated by the latter [8]. Furthermore, the high costs associated with medical care and the aging population strongly stress the need to expand our knowledge base on all aspects of PD [13]. The various effects of which NMS are comprised and their highly divergent patterns of progression between PD patients further raise the challenge imposed by NMS in the management of PD [15].

About a decade ago, Dr. Braak et al. proposed the intriguing hypothesis that PD might result from an infection spreading first by intestinal and olfactory mucosae [22, 23]. This proposal followed the first description of Lewy bodies in the dorsal vagal nucleus by Friederick Lewy in the early 20th century [6, 15]. Based on Lewy bodies distribution in PD postmortem patients, Braak et al. also suggested six neuropathological stages, corresponding to disease evolution [23]. As such, the first signs of Lewy pathology appear in projection neurons of the dorsal motor nucleus of the vagus nerve at the early stage of PD [23]. Despite its potential interest, this hypothesis is not widely accepted, mainly because of the paucity of patients studied and the lack of associated clinical data [24]. However, the manifestation of NMS, preceding motor diagnosis, closely corresponds to the progression of Lewy pathology, supporting Braak's hypothesis [8]. Some studies have further suggested that the pathological process leading to PD could be initiated in the enteric nervous system (ENS) before spreading to the central nervous system (CNS) via autonomous connections such as through the vagus nerve [25, 26]. In connection with the latter observation, a recent study has demonstrated that different forms of human alpha-synuclein (*α*-syn), the major protein component in Lewy bodies, injected in the intestine of mice can propagate to the brain via the vagus nerve and reach the dorsal motor nucleus in the brainstem, supporting Braak's hypothesis [27].

There are several different approaches to categorize the nonmotor features encountered in PD, but they have usually been separated into five major classes, namely, cognitive impairment, neuropsychiatric disorders, autonomic dysfunction, sleep disturbances, and other NMS [4–7]. Confusion and dementia are the most commonly reported cognitive impairments, whereas neuropsychiatric disorders rather occur as hallucinations, anxiety, depression, and impulse control disorders. Importantly, PD medication can potentially exacerbate some of the latter problems [13]. For example, the effects of DA agonists on the mesolimbic pathway could be responsible for impulse control disorders such as compulsive gambling, compulsive shopping, and hypersexuality [7, 28]. In addition, an injury to the autonomic nervous system can be observed in various peripheral NMS such as orthostatic hypotension, functional bladder disorder, excessive sweating, erectile dysfunction, and gastrointestinal (GI) symptoms such as constipation, drooling, dysphagia, and nausea [4, 6, 13, 16, 26, 28]. Other nonmotor features that are still poorly categorized include pain, fatigue, unexplained weight changes, and visual as well as olfactory disturbances. To better identify these elements, Chaudhuri et al. developed the Non-Motor Symptoms Scale, which allows for a more accurate measurement of the frequency and severity of NMS and allows determining the impact of treatment on these symptoms [15, 29]. In addition, the Non-Motor Questionnaire, the Scales for Outcomes in Parkinson's Disease, and a revised version of the UPDRS (sponsored by the Movement Disorder Society) also contribute to the establishment of standardized and reliable means to assess NMS in PD [8, 30].

## 2. GI Manifestations in Autonomic Disorders

Early PD, when left untreated, is often accompanied by autonomic nervous system impairments among which GI symptoms represent the most common NMS [31]. Indeed, several studies relying on nonmotor rating scales have underscored the particular significance of GI symptoms in assessing the quality of life and have shown that these manifestations occur in 60% to 80% of patients [13, 16, 32, 33]. GI disorders are among the most common causes of emergency admission and often result in severe complications such as malnutrition (15% of PD patients), pulmonary aspiration (2.4% of PD patients), megacolon (mostly asymptomatic; incidence unknown), intestinal obstruction (rarely reported; incidence unknown), and even intestinal perforation (a few cases reported; incidence unknown) [34–38]. Moreover, older age, DAergic medication, and higher disease severity are usually associated with these nonmotor features [28]. Hence, GI symptoms reflect disturbances of GI tract motility at all levels.

There are two major neural influences that regulate the GI tract, namely, the extrinsic pathway, which is associated with the vagus nerve, and the ENS, a component of the autonomic nervous system [39]. Due to its capacity to operate independently of the CNS and its 100 million neurons, the ENS is often considered as the second brain of the human body [39–41]. The ENS contains the myenteric and submucosal plexi, which are responsible for controlling smooth muscle activity in the GI tract [40, 41]. The latter intestinal function, which is regulated by the ENS, requires the involvement of several types of neurotransmitters such as DA, serotonin, acetylcholine, vasoactive intestinal peptide (VIP), substance P, and nitric oxide synthase (NOS) [42]. Although the ENS has the ability to function independently of external stimuli, it also closely interacts with the vagal system [39, 41].

### 2.1. Constipation

Constipation is one of the initial NMS related to PD pathophysiology, affecting about 50–80% of patients. It often occurs early in the course of the disease and may precede the appearance of motor symptoms by several years [6, 13, 28, 31, 43, 44]. Constipation is usually defined as fewer than three bowel movements per week and straining to pass stools [45]. Although constipation is mainly considered as a delay of the GI transit, some evidence suggests that it can also be ascribed to a paradoxical contraction of voluntary sphincters during defecation, resulting in difficulties with rectal expulsion. In the early stages of PD, decreased GI motility has been associated with neuronal loss in the myenteric and submucosal plexi and inclusions of Lewy bodies in the dorsal motor nucleus of the vagus, underscoring their potential role in slowing down intestinal peristalsis [7, 28, 32]. In addition to its association with autonomic alterations and, in some cases, urologic impairment, constipation is linked to a 2.7- to 4.5-fold increase in the risk of suffering from PD [15, 43, 46]. Constipation may also be accompanied by other GI features that can affect intestinal transit. For instance, pain, nausea, bloating, vomiting, and distension are all symptoms of paralytic ileus, inducing complete obstruction of the gut and affecting about 7% of parkinsonians. Anismus, the abnormal contraction of the external anal sphincter and puborectalis muscle during attempted defecation, is another problem that can occur in synergy with constipation in approximately 65% of PD patients, which is more frequently observed during “off” periods [16, 28, 47]. Other intestinal complications such as megacolon (mostly asymptomatic), pseudoobstruction, sigmoid volvulus, and bowel perforation may also arise in severe conditions, although their exact incidence is still currently unknown [32, 37, 38, 48].

### 2.2. Drooling

Also known as sialorrhea, drooling is the most common NMS of PD and is generally predominantly observed in the late stages of the disease and during the “off” state medication [5, 49, 50]. Affecting 70 to 80% of parkinsonians, sialorrhea corresponds to an exaggerated increase of saliva production and/or retention in the mouth cavity, with occasional overflow into the pharynx [13, 32, 49–51]. The submandibular, sublingual, and parotid glands are the three pairs of salivary glands responsible for most of the approximately 1.5 liters of saliva secreted daily and are controlled by the autonomic nervous system, mainly under parasympathetic cholinergic innervations [52, 53]. Sialorrhea may result from three phenomena, namely, abnormal production of saliva, impairment of salivary clearance, and/or inability to maintain saliva in the mouth [51]. Furthermore, excessive salivary production may sometimes lead to serious complications, including saliva-induced asphyxiation and aspiration pneumonia [31, 45]. Different scales, such as Drooling Severity and Frequency Scales, Drooling Rating Scale, and Sialorrhea Clinical Scale for PD, have been proposed to assess sialorrhea according to standard criteria [52, 54, 55]. However, drooling is rarely due to overproduction of saliva but is rather more common due to dysphagia, which itself is essentially a manifestation of bradykinesia [50, 56]. Indeed, in most PD patients, decreased salivary production is in fact observed [51, 56, 57]. Studies have shown that patients do not produce excessive amounts of saliva but rather have a more limited ability to swallow properly which, when associated with a forward head posture, might contribute to the onset of drooling [32, 49, 58]. In general, the inability to control oral secretions can affect eating and speech and cause social embarrassment [59]. Some patients even consider sialorrhea as their worst PD symptom [32]. Different factors can influence sialorrhea, such as male gender [60], aging [61], severity and duration of PD [62], hallucinations [59], orthostatic hypotension, dysphagia, dysarthria, UPDRS scores, and the use of antidepressants [51, 63]. Furthermore, the peripheral autonomic nervous system and the dorsal motor nucleus of the vagus nerve have been implicated in drooling, and Lewy bodies have been found in the submandibular salivary glands in some studies [5, 64].

### 2.3. Dysphagia

Dysphagia, a feature of PD pathophysiology, is defined as a difficulty in swallowing food, liquids, or pills due to an impaired function of the medullary center [65, 66]. Dysphagia can result from muscular coordination dysfunctions in at least one of the three phases of deglutition: oral, pharyngeal, and oesophageal [67]. The main cause of swallowing difficulties, that is, a dysfunction of the oropharyngeal phase (found in about one-third of PD patients [68]), often results from motor symptoms of bradykinesia and a reduced motor control of the tongue. Thus, these motor features contribute to the pathophysiological development of dysphagia and, by extension, might also play a role in the onset of sialorrhea in PD [51]. Various abnormalities in the oropharyngeal phase, such as a delayed swallowing reflex, laryngeal movement deficits, and vallecular and piriform sinus residues, have been reported [66, 69]. In the oesophageal phase, complete aperistalsis, simultaneous oesophageal spasms, slower oesophageal transit, and deficit in sphincter relaxation and pressure have been the predominantly observed abnormalities [67, 70]. Interestingly, this involuntary component of deglutition is under autonomic control, and Lewy bodies have been identified in the oesophageal myenteric plexus [66, 67]. These findings suggest that swallowing impairment could partly result from direct damage to the ENS. Moreover, in view of the various aforementioned abnormalities, dysphagia is clearly linked to an increased risk of mortality by causing and/or exacerbating other PD-related complications such as aspiration pneumonia (estimated to account for 70% of the mortality rates among PD patients [36]), choking, malnutrition, unexplained weight loss, and dehydration [13, 66, 69, 71]. Unfortunately, the degree of dysphagia cannot be predicted by PD progression because it has no direct connection with the clinical severity of the disease as evaluated by motor criteria [31, 70]. Moreover, data from various studies suggest that up to about 50% of parkinsonians might suffer from deglutition problems, which, as with drooling, occur mainly during the late stages of the disease [66, 71, 72].

### 2.4. Nausea, Vomiting, and Gastroparesis

Nausea and vomiting (which are experienced by approximately 20% of patients [45]) are related, most of the time, to antiparkinsonian medications for motor symptoms, rather than occurring as intrinsic features of PD [6, 7, 28]. Indeed, these side effects generally appear following the initiation of DAergic treatments [28]. However, nausea may likely occur in untreated parkinsonian patients as well, and such cases might be explained by underlying gastroparesis [73]. Also known as delayed gastric emptying, gastroparesis corresponds to decreased stomach motility, which may eventually affect gut transit. In addition to nausea, chronic gastroparesis is characterized by early satiety, a sensation of fullness, weight loss, and abdominal pain and bloating [74]. This phenomenon could well be related to the degeneration of autonomic neurons in the myenteric plexus and brainstem [45]. Moreover, intestinal absorption of L-DOPA and other medications might be slowed by such protracted gastric retention, thus reducing the effectiveness of treatment and preventing the improvement of motor symptoms [75]. PD-associated gastroparesis deserves proper medical attention as its observed prevalence approaches 90% of patients [76].

### 2.5. Pathophysiology

Recently, several clinical and postmortem studies exploring Lewy bodies expression and/or the presence of neurodegeneration in the enteric nervous system of parkinsonian patients have been conducted in order to better understand the etiopathogenesis of PD (see Table 1).

#### 2.5.1. Lewy Bodies

The pathophysiological mechanisms underlying GI dysfunctions are likely to be multifaceted, reflecting not only the involvement of the intrinsic innervation of the gut, but also extrinsic inputs because of the presence of Lewy pathology in the dorsal motor nucleus of the vagus, sacral parasympathetic nuclei, and sympathetic ganglia [77–79]. The occurrence of Lewy pathology in the gut of PD patients was first reported in an autopsy survey in which Qualman et al. found myenteric Lewy bodies in the colon of one patient and in the esophagus of another [80]. A subsequent clinical study demonstrated the presence of Lewy bodies in the colon of one PD subject [81]. These primary observations led Wakabayashi et al. to perform a systematic assessment of Lewy pathology in the ENS of several PD patients [82]. Lewy bodies were found in the GI tract of seven patients and were distributed widely from the upper esophagus to the rectum. In a follow-up study, the same team reported that most Lewy bodies observed within the GI tract of the three patients were located in VIP^+^ neurons and to a lesser extent in neurons immunoreactive for tyrosine hydroxylase (TH) [83]. Therefore, this suggests potential interplay between these neurons and cholinergic neurons of the vagus nerve contributing to the spread of *α*-syn to the CNS. It was also mentioned that few Lewy bodies were found in neurons that were negative for either VIP or TH. To date, these have been the only studies suggesting that a specific subset of enteric neurons could bear Lewy pathology [83]. No further reports regarding GI Lewy pathology in patients with PD were published, until 2006 when Braak et al. brought this topic to the forefront [84]. In this postmortem study, they investigated the gastric myenteric and submucosal plexi from five individuals with Lewy body disease. Clinical data demonstrated that three out of the five patients with Lewy body pathology displayed motor symptoms reminiscent of PD while the other two patients were reported to be free of such symptoms. However, Lewy pathology was present in both the myenteric and the submucosal plexi of all five patients. This led Braak and colleagues to postulate that the pathology initiates in the ENS before progressing to the CNS [84]. Despite being a potentially important finding, this hypothesis has not been widely accepted, mainly because of the paucity of patients studied and the lack of associated clinical data [24]. More recently, a comprehensive survey on the occurrence of Lewy pathology in the peripheral nervous system, and especially in the ENS, has been published by the Arizona Parkinson's Disease Consortium [79]. One of the most striking results of this study was the identification of Lewy inclusions in the esophagus of 14 out of 15 PD patients, suggesting that enteric pathology is present in the vast majority of PD cases [79]. Other recent studies have also observed *α*-syn positive staining in GI tissues collected before patient's diagnosis [85] and in the vast majority of parkinsonian patients' colon tissues [86, 87].

The abovementioned data on the ENS in PD patients were collected either at autopsy or using colectomy specimens. To extend this work by analyzing enteric neuropathology in living patients, Lebouvier et al. took advantage of a novel colonic biopsy technique [88, 89]. Twenty-nine patients with an established PD diagnosis were enrolled together with 10 healthy subjects who had undergone colonoscopy for colorectal cancer screening. Biopsies from 21 out of the 29 patients with PD (72%) showed Lewy neurites in their submucosal plexus, whereas no Lewy pathology was observed in any of the controls [89]. Chronic constipation was more frequent in patients with than without Lewy neurites, suggesting a pathogenic role for these inclusions. However, Lebouvier et al. did not consider the myenteric plexus, which is directly involved in the control of bowel motility [89]. These findings are in line with other reports on PD enteric pathology, which showed that, besides Lewy bodies, Lewy neurites were also observed in the ENS of patients [79, 84, 90–93]. Using *α*-syn immunostaining, the authors also demonstrated that approximately half of the Lewy neurites observed in the submucosal plexus belonged to postganglionic neurons, thus supporting their extrinsic origin [84]. The origin of the remaining Lewy neurites remains to be determined, but it is possible that they could originate both from submucosal and from myenteric neurons, which have been shown to project to the submucosal blood vessels [94]. This observation is in agreement with recent studies showing *α*-syn immunolabeling in the submucosal perivascular regions [95, 96]. Depending on the type of *α*-syn immunostained and the intestinal region studied, some discrepancies in the observation of Lewy bodies in GI biopsies or postmortem tissues are possible, especially because *α*-syn is physiologically expressed by red blood cells and vascular endothelial cells [96].

Interestingly, an animal model of PD recently developed provides some clues on the role of ENS alterations in GI dysfunction. Transgenic *α*-syn SNCA, A53T, and A30P mice display aggregates within their enteric ganglia, which is associated with a prolonged whole-gut total transit time and reduced colonic motility [97]. However, there is no evidence of pathologic changes in the dorsal motor nucleus of the vagus or autonomic cardiovascular dysfunction. These findings suggest that ENS alterations in these mice are intrinsic in origin, being caused by *α*-syn aggregation in enteric neurons only. It is possible in PD patients that at least some of the GI symptoms could be caused by enteric neuropathy. It should be pointed out, however, that studies on GI symptoms in PD have focused mainly on motility disorders and therefore the role of the myenteric plexus and associated consequences of Lewy pathology in the submucosal plexus have, to our knowledge, not been addressed either in patients or in experimental models of PD.

#### 2.5.2. Neurodegeneration

Enteric neurons produce a substantial amount of DA which regulates normal gut motility [67]. Interestingly, slowed GI transit and decreased gut contraction in PD patients occur via altered DA-ENS circuitry, which normally promotes the peristaltic reflex [98]. PD patients with severe constipation have been reported to present lower levels of GI DA, suggesting that damage to the enteric DAergic system might be an important factor underlying GI dysfunction [99]. More recently, age-related loss of myenteric neurons has been associated with chronic constipation, although studies are widely controversial [100, 101]. Unfortunately, it is still not clear whether PD leads to the loss of enteric neurons. Singaram et al. reported that most patients present DAergic neuronal loss in the colonic myenteric and submucosal plexi, whereas other types of neurons were not affected based on TH immunostaining [99]. Other teams also used this marker on postmortem tissues and colon biopsies, and none reported DAergic enteric neuronal loss [88, 92, 102, 103].

Systemic administration of the selective DAergic neuronal toxin 1-methyl 4-phenyl 1,2,3,6-tetrahydropyridine (MPTP) leads to the loss of DAergic neurons in the intestinal tracts of mice [104, 105], but MPTP-treated monkeys were reported to display an increased number of neurons in their myenteric ganglia [106]. MPTP causes a transient increase of stool frequency and colon relaxation lesions in mice [104], although this effect is inconsistent with the slow GI motility of PD patients. Therefore, despite the fact that inhibitory intestinal DAergic neurons could be impaired in PD, these neurons are not the only neuropathological targets of the disease [106–108]. Indeed, intestinal non-DAergic neurons could also be impaired, but the discrepancy between data makes it difficult to draw robust conclusions. Anderson et al. demonstrated that MPTP-treated mice presented no difference in nitric oxidergic neurons [104]. Another study showed in a PD model induced by directional stereotaxic brain injection of the neurotoxin 6-hydroxydopamine (6-OHDA) that rats exhibited slow colon motility accompanied with nitric oxidergic neuron loss in the myenteric plexus [109]. Other studies showed that a primate MPTP model led to an increase in nitric oxidergic neurons [106]. Overall, most of these studies have shown that GI cholinergic transmitters were not significantly altered in PD [104, 106, 110].

According to these data, constipation in PD patients cannot be explained solely by a decrease in DA levels linked to damage to neurons. Digestive tract motility would require sophisticated synchronization from all neurotransmitters, not only DA. Moreover, the important variability between the results pertaining to enteric neuronal loss refers to the neurodegenerative paradox. Even if DAergic neuronal death is the histopathological hallmark of PD, it is one of the most difficult parameters to highlight in the ENS because of both the rarity of apoptosis in the neurodegenerative process and the difficulty in counting neurons [111]. This has together led to numerous unanswered questions concerning neurodegenerative processes occurring in the ENS and their impact on GI impairments.

### 2.6. Other Outcomes of PD Therapies on GI Dysfunctions

Antiparkinsonian medication considerably hampers the evaluation of the potential correlation between GI dysfunctions and the severity of PD symptoms. An individual stabilized by drug therapy may indeed display a better overall condition than another patient with early PD, thus receiving a suboptimal treatment [37]. Moreover, in some situations, addressing motor symptoms only may affect GI features both positively and negatively. Indeed, DAergic therapy may improve dysphagia and drooling but, on the other hand, might also worsen gastroparesis and reduce GI motility [69, 75, 112]. However, since nausea and vomiting are often side effects of various medications, they can limit the use of the latter and, as a result, preempt the benefit of such medications on motors symptoms [31]. Moreover, deep brain stimulation (DBS), which is widely used to treat motor symptoms, has been shown to have a potential impact on the manifestation of GI symptoms [113, 114]. According to some studies, constipation and deglutition are significantly improved after surgery in the subthalamic nucleus [115–117]. However, there is no consensus on the putative effect of DBS on GI manifestations, as shown by reports that the latter neurosurgery does not improve dysphagia and drooling [51, 118, 119].

## 3. Therapeutic Approaches to GI Symptoms

**Figure 1:**
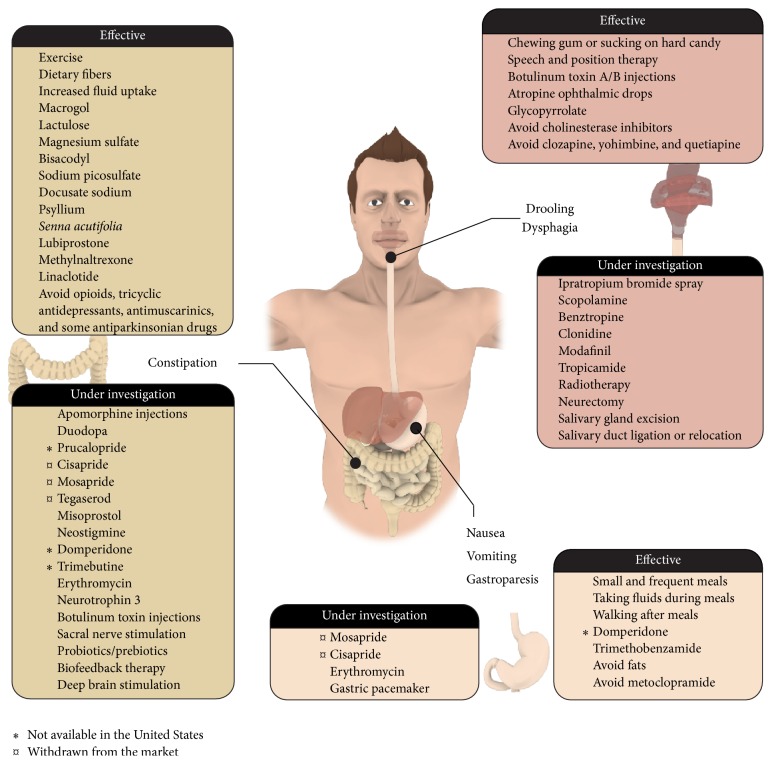
Treatment options for gastrointestinal dysfunctions in Parkinson's disease. Overview of the different pharmacological treatments or therapeutic approaches that are currently effective or under investigation to manage constipation (left panels), drooling/dysphagia (right panels), and nausea/vomiting/gastroparesis (bottom panels). Please note that some drug options are not available in the USA (*∗*) or had to be withdrawn from the market due to unacceptable side effects (*¤*).

Importantly, GI impairments can impact other symptoms, which further complicates the clinical management of PD. For instance, GI problems such as gastroparesis and delayed intestinal absorption might lead to more erratic absorption of L-DOPA, which is reflected by motor fluctuations [120]. The latter problem emphasizes the necessity for clinicians to exert due vigilance during office visits of PD patients and regularly ask specific questions regarding GI manifestations. Recent studies have also provided evidence for symptomatic treatments of constipation and drooling, but, unfortunately, the current armamentarium for dysphagia and nausea remains quite limited [31]. In this regard, Figure 1 and Table 2 provide a summary of GI symptoms as well as the current treatment alternatives.

### 3.1. Constipation

#### 3.1.1. Effective Treatments

To prevent constipation problems in PD, therapies aimed at accelerating colonic transit may be effective. Increasing the levels of daily activity and introducing dietary changes are the first options to consider. Patients should be encouraged to maximize dietary fibers (cereals, bran, citrus fruits, etc.), as well as ensure adequate fluid intake to avoid dehydration [6, 15, 16, 32]. Nevertheless, an exhaustive pharmaceutical evaluation of the drug treatments already prescribed to patients is important before introducing additional measures. Indeed, the dosage of medications known to increase constipation symptoms should be optimized as much as possible. Some antiparkinsonian drugs as well as opioids, tricyclic antidepressants, and antimuscarinics are recurrent sources of severe constipation, likely due to their anticholinergic effects [15, 52]. Other available options to increase the frequency of bowel movements and improve stool consistency are (i) osmotic laxatives such as macrogol (polyethylene glycol), lactulose, and magnesium sulfate, (ii) stimulant laxatives such as bisacodyl and sodium picosulfate, and (iii) stool softeners [28, 30, 121–123]. The safety profile associated with the long-term use of osmotic agents makes them the preferred group of laxatives. Macrogol, which is available in the USA and is recommended by the American Academy of Neurology and the Movement Disorders Society, is considered to be an effective and safe osmotic laxative for PD patients [15, 32, 121]. Bisacodyl and sodium picosulfate, which both act by stimulating colonic smooth muscle contractions as well as electrolyte and water secretion, may represent additional alternatives to treat constipation [124]. Moreover, stool softeners such as docusate sodium may be used alone or in combination with psyllium husks to increase stool volume and, therefore, peristalsis reflex [6, 7, 125]. By increasing intestinal fluid secretion, lubiprostone, an intestinal ClC-2 chloride channel activator, also improves constipation issues (64% of PD patients) [7, 28, 52, 126]. The most common adverse events observed were intermittent loose stools (48% of PD patients), nausea (29%), diarrhea (12%), abdominal pain (8%), flatulence (6%), dizziness (3%), and vomiting (3%) [52, 126, 127]. Methylnaltrexone (*µ*-opioid antagonist) is another medicinal agent approved in the USA and indicated for the treatment of opioid-induced constipation, with approximately 60% of patients having reported beneficial intestinal effects [28, 128]. In 2008, a clinical trial led by Portenoy et al. showed that adverse effects experienced by patients taking methylnaltrexone are mostly abdominal pain (45%), flatulence (33%), diarrhea (30%), and nausea (24%) [128]. Linaclotide, a guanylate cyclase C agonist, has also recently been approved by the Food and Drug Administration (FDA) as a treatment for irritable bowel syndrome and chronic constipation. Abdominal cramping, discomfort, and diarrhea are the adverse events commonly reported by patients for linaclotide (about 4%) [52, 129, 130]. Finally, several other studies have also demonstrated the effectiveness of the* Senna acutifolia* plant, but the long-term use of this well-known laxative is not recommended [122].

#### 3.1.2. Treatments under Investigation

Treating constipation remains an active research area and various studies have assessed the impact and clinical relevance of options that could help relieve the discomfort and adverse effects associated with this GI problem encountered in PD. For example, subcutaneous injections of apomorphine have translated to positive effects on intestinal motility (improvement of the defecatory mechanisms and anorectal dysfunction [6, 32, 131, 132]) and UPDRS motor scores (in about 70% of PD patients [133]), although adverse effects such as orthostatic hypotension (in 50% of patients), nausea, and drowsiness (in 75% of patients) may occur following administration of this DA agonist [8, 134]. It is also recommended that patients use an antiemetic as a pretreatment before receiving injections in order to avoid the unpleasant effect of nausea [31]. Therefore, due to these various secondary effects, the long-term use of apomorphine appears to be inadvisable [32]. Intrajejunal infusion of L-DOPA/carbidopa (or duodopa) has also proved beneficial relatively to constipation problems (in approximately 70% of PD patients) [135, 136]. Moreover, a body of research has been heavily focused on different ligands (agonists or antagonists) of the 5-HT_4_ serotonin receptors. These receptors, which are located partly in the smooth musculature and cholinergic nerves of the GI tract, are, among others, capable of increasing gastric and colonic motility by facilitating acetylcholine release [137–139], thus making them an attractive target for treating constipation. The main 5-HT_4_ agonists studied to date are prucalopride, cisapride, mosapride, and tegaserod [137, 140–142]. Unfortunately, although these agonists were found to be effective in the treatment of constipation in PD patients, those prokinetic agents have been removed from the US market or have not been approved by the FDA due to possible adverse cardiovascular effects (less than 1% of patients) [141, 143–145]. Other medicinal agents are also under investigation, such as misoprostol (a prostaglandin E_1_ analogue; 55% efficacy) [32, 146], neostigmine (an acetylcholinesterase inhibitor; 50% efficacy) [7, 147], and domperidone (a DA antagonist; about 35% efficacy) [148]. However, even if the promotility agent domperidone could be potentially effective, due to its absence of permeation through the blood-brain barrier (BBB) [149], there is insufficient evidence to recommend its utilization for constipation, as in the case of trimebutine (an enkephalinergic agonist) and erythromycin (the well-known macrolide antibiotic) [143]. In recent years, the NGF receptor agonist neurotrophin 3 has also been studied to improve GI motility dysfunction in PD. Although its mechanism of action with respect to GI motility remains unknown, this neurotrophic factor was found to be effective in treating constipation (in about 20% of patients) [52, 150]. In a clinical trial conducted by Pfeiffer et al., a reduced colonic transit time, an increase in stool frequency, and shortening of the intervals without stool were observed [151]. However, abdominal cramps and diarrhea were noted in three patients, who were forced to reduce neurotrophin 3 dosage (300 *µ*g/kg three times weekly) [151]. Injections of botulinum toxin (BTX), a neurotoxin produced by the* Clostridium botulinum* bacterium that inhibits acetylcholine release, have also been proposed to help reduce constipation burden in PD [51, 152]. However, not only are such injections technically challenging, including ultrasound guidance, but also there is insufficient evidence that this method offers an effective treatment [30, 153, 154]. For example, Albanese et al. reported a beneficial clinical effect of BTX injections on constipation, but only in a single patient [153]. In another clinical study, Cadeddu et al. observed an improvement of constipation symptoms in 10 out of 18 patients after two months of BTX treatment [154]. However, the authors mentioned that repeated injections could be necessary to maintain this clinical improvement since the effects of the toxin wear off within three months of administration. Nonpharmacological strategies have also been put forward to treat constipation, such as sacral nerve stimulation (with 57% efficacy) [28, 155], synbiotic yogurt (i.e., probiotic- and prebiotic-enriched yogurt) [7, 16, 52], biofeedback therapy (79% efficacy) [52], and DBS (about 25% efficacy after two years of treatment, a percentage that might however be influenced by the postoperative reduction in DAergic therapy and an improvement in motor fluctuations) [116, 117]. Milk fermented with the probiotic strain* Lactobacillus casei* Shirota has also been suggested to dampen constipation problems by modulating the host immune response, enhancing mucosal function, suppressing growth of pathogenic bacteria, and blocking epithelial attachment by pathogens, resulting in an improvement in 70 constipated adults [156, 157]. A decrease in abdominal pain, bloating, and sensation of incomplete emptying is also observed in patients using probiotics [52].

### 3.2. Drooling and Dysphagia

#### 3.2.1. Effective Treatments

For patients with mild symptoms of drooling and/or dysphagia, chewing gum or sucking on hard candy may be effective in ameliorating swallowing (an approximately 5-fold improvement) and thus reduce drooling [13, 67, 152, 158]. Speech and position therapies can also prove efficient for easing these GI symptoms (with 60 to 90% efficacy) [159]. These therapies consist basically in training to learn voluntary airway protection techniques through adequate swallowing methods and improved head postures. Marks et al. investigated such techniques and observed that self-motivation was an important factor in obtaining a positive outcome [160]. It is strongly recommended to consider all these nonpharmacological options first to improve drooling and dysphagia symptoms before changing over to drug-based solutions. However, such drug-free approaches may only provide temporary improvement and might not be effective or suitable for all patients. Indeed, pharmacological treatments are generally considered when more aggressive intervention is required [31]. It must be emphasized that some categories of medications used to treat other PD symptoms may in fact aggravate drooling and dysphagia and should thus be avoided as much as possible. Such medications include acetylcholinesterase inhibitors, the antipsychotic quetiapine, and adrenergic receptor agonists such as clozapine and yohimbine [51, 161, 162]. The pharmacological treatment most often mentioned for drooling/dysphasia is undoubtedly BTX injections. Local injections of this toxin in the parotid and submandibular glands inhibit the cholinergic parasympathetic and postganglionic sympathetic activity, thereby reducing saliva production [163]. This treatment, which denervates the salivary glands, was shown to be effective in reducing drooling severity and frequency (in about 80 to 90% of patients) without compromising swallowing [50, 51, 164–167]. Unfortunately, published studies cannot be easily compared due to the important disparity between the methodologies employed. Indeed, there is no standard technique for the injection (gland, ultrasound guidance, etc.) and no compliance regarding the optimal dose to be administered [51]. The sole guideline for achieving the best effect using this therapeutic approach is to inject the toxin bilaterally and periodically [31, 163]. Dryness of the mouth (or xerostomia) is the common adverse effect observed with BTX [51]. Importantly, submandibular glands injections are recommended only under the supervision of a specialist due to potential side effects caused by spreading of the toxin to nearby structures and should be performed exclusively when treatment of the parotid gland alone is insufficient [163]. Among the several serotypes of BTX, only A and B have been studied and are commercially available [51]. In the majority of these studies, no side effects were observed with BTX-A, although BTX-B injections induced mild adverse events such as dry mouth (in about 40% of patients), diarrhea (~15%), neck pain (~15%), and worsened gait (~25%) [50, 168, 169]. This suggests a preferential action of BTX-B on autonomic neurons and therefore might point to its higher effectiveness compared to BTX-A [152]. However, in two different clinical trials, Lagalla et al. observed that some patients experienced mild transitory swallowing difficulties 7 days after a BTX-A injection (in about 6% of patients) [166] and 10 days after a BTX-B injection (~16%) [167], but they recovered within 10 to 14 days. In spite of these potential drawbacks, these studies, which are the only ones that have compared the A and B serotypes, failed to demonstrate a significant difference in the effectiveness between both neurotoxins [166, 167]. Other pharmacological alternatives to BTX in the treatment of drooling/dysphagia include anticholinergic drugs that block muscarinic receptors and particularly the M3 subtype. Nevertheless, the currently available agents are not selective for M3 receptors and might thus give rise to several undesirable side effects (e.g., confusion, hallucinations, drowsiness, urinary retention, and constipation) [51]. Thus, some of these drugs have yet to be considered truly effective, which warrant further investigations. A few studies have claimed that the two anticholinergics, atropine and glycopyrrolate, are the only potentially useful therapies available for improving drooling/dysphagia [51, 52, 123]. Despite being effective, atropine still causes a wide range of undesirable adverse effects such as hallucinations (2/7 patients) and delirium (1/7 patients; but this was confounded by a concomitant urinary tract infection) [170]. Since glycopyrrolate does not cross the BBB, unlike atropine, it is therefore the preferred agent because it is less likely to cause adverse effects in the CNS [152, 171]. Between 95 and 100% of patients who completed clinical studies reported improvement in drooling/dysphagia with glycopyrrolate [172–174]. As expected, the side effects observed occurred in the periphery and mostly included xerostomia (in approximately 52% of patients), urinary retention (13%), constipation (13%), vision problems (13%), and nausea (~4%) [171, 175]. While anticholinergics might be efficient for treating drooling/dysphagia, they do not represent a suitable option for PD patients since other NMS can be subsequently worsened. Moreover, there is a lack of clinical evidence for treatments lasting longer than a few weeks and the long-term adverse effects of atropine and glycopyrrolate have not been documented, thus leaving important safety issues unresolved [51, 171]. All the pharmacological options listed above may thus be regarded as effective treatments for drooling/dysphagia, but, considering their potential side effects, they should remain a secondary choice compared to nonpharmacological therapies.

#### 3.2.2. Treatments under Investigation

Other anticholinergic treatments such as ipratropium bromide spray, transdermal scopolamine, and benztropine have also been investigated for treating drooling/dysphagia [123, 176–178]. However, previous studies on the effectiveness of anticholinergic treatments had failed to conclude on the superiority of one drug over another [179]. The ipratropium bromide spray (which has induced a significant effect on the UPDRS part 6 subscore [178]) is used sublingually as a bronchodilator and does not cross the BBB, thereby reducing systemic side effects [152]. Unfortunately, there is insufficient data about its safety and efficacy to draw definite conclusions on its potential interest in drooling/dysphagia management [51, 123]. Adrenergic receptors agonists have also been explored in this context. Clonidine, a selective *α*2-adrenergic receptor agonist, significantly improved the frequency at which patients had to clear their mouths [51, 52, 152]. The most common adverse events observed with clonidine were diurnal somnolence (2/17 patients), dizziness (1/17), and dry mouth (1/17) [180]. The *α*1-adrenergic agonist modafinil has also been reported to exert rather beneficial effects on drooling/dysphagia in PD patients (6/6 patients), although this improvement was mostly related to dysphagia rather than hypersalivation [51, 181]. Moreover, Lloret et al. have investigated tropicamide, a short-acting muscarinic receptor antagonist, in the treatment of drooling/dysphagia. So far, this treatment has shown potential efficacy (33% average reduction in salivary volume for 16 patients who completed the study) along with a lack of noticeable side effects and no side effects, although the data must still be considered as preliminary [182]. Radiotherapy has also been suggested as a treatment for drooling/dysphagia and studies in this context have shown a significant improvement in symptoms (79% of patients), an effect that could be maintained for at least one year [183]. Common side effects were xerostomia (40% of patients) and a loss of taste (45%), which were mostly transient (25% and 35%, resp.). Regrettably, the success of radiotherapy is largely compromised by its potential to induce neoplasia [183, 184]. Therefore, this treatment should only be considered when all other options discussed above have proved ineffective. Finally, surgical options such as neurectomy, salivary gland excision, salivary duct ligation or relocation, and DBS have also been explored to ameliorate drooling/dysphagia [50, 118, 184–187]. Neurectomy, that is, the surgical sectioning of the chorda tympani nerves, reduces salivary production (improvement in 74% of patients) but might induce serious complications such as hearing loss and a loss of taste [152, 188]. These invasive options (neurectomy and salivary gland/duct surgeries) can be realized individually or in combination (with >75% success) and possible adverse effects include dental caries (10% of patients), cracked lips (10%), aspiration pneumonia, and xerostomia [152, 184–186]. Due to their high risk of irreversible adverse effects, all these interventions are considered only when all other available options have failed to bring about a positive outcome [32]. DBS intervention has not been studied much to date in the context of drooling/dysphagia improvement, but, with the limited information obtained so far, it seems unlikely that DBS represents a useful option [51, 118].

### 3.3. Nausea, Vomiting, and Gastroparesis

#### 3.3.1. Effective Treatments

Despite substantial progress in recent research on constipation and drooling treatment, the armamentarium of useful agents for other PD-associated GI symptoms, such as nausea, vomiting, and gastroparesis, remains severely limited [31]. The effective antiemetic medications that have been investigated so far include domperidone (100% efficacy) and trimethobenzamide (~20% efficacy) [123, 198, 199]. Domperidone is a peripheral DA antagonist that does not cross the BBB and has been reported to safely improve gastroparesis and associated GI symptoms in PD patients [199]. This antiemetic agent is not available in the USA but is prescribed in many other countries across the world [13, 16]. Interestingly, metoclopramide, another DA receptor antagonist often employed in gastroparesis treatment, is contraindicated for PD patients because it worsens motor symptoms by blocking DA receptors in the CNS [31]. Finally, changes in the lifestyle of patients with nausea, vomiting, and gastroparesis symptoms are also strongly recommended. Thus, having small and frequent meals, avoiding high-fat foods, drinking during meals, and walking after meals are the suggested options [31].

#### 3.3.2. Treatments under Investigation

Other treatments have been considered to improve nausea, vomiting, and gastroparesis in PD patients. Mosapride and cisapride, two mild 5-HT4 serotonin receptor agonists that act as prokinetic agents, have been shown to reduce gastroparesis symptoms in PD (3/5 and 8/12 patients, resp.) [200, 201]. However, due to their cardiotoxicity, these drugs have been removed from the US market [31]. Other potential options such as erythromycin and the implantation of a gastric pacemaker might be beneficial to correct gastroparesis, but they have not yet been specifically tested in PD patients [31]. Furthermore, electric stimulation, surgery, or application of BTX in the pyloric sphincter can be employed, albeit exclusively in extreme cases [16].

### 3.4. Possible Interactions of PD Treatment with GI Dysfunctions

As mentioned above, treatments for motor symptoms may influence GI symptoms, but the opposite may also hold true [31]. These considerations hamper interpretations as to whether symptoms observed in a given patient reflect the disease* per se* or, on the contrary, are iatrogenic. For instance, L-DOPA is usually administered in combination with carbidopa, which is well known to exacerbate nausea [13]. In the periphery, carbidopa prevents the conversion of L-DOPA to DA, and as its half-life exceeds that of L-DOPA, one might theoretically expect residual effects of carbidopa outside the CNS [202]. This treatment might well prevent the conversion of endogenous peripheral L-DOPA in addition to the exogenous L-DOPA that is concomitantly administrated. Such potentially protracted effects of the combination therapy due to putative residual carbidopa could result in decreased DA production in the periphery, which would then affect NMS, including GI features. It has also been shown that carbidopa might influence DA concentrations in the kidney [203]. Therefore, the potential impact of carbidopa on peripheral organs involved in NMS deserves careful evaluation. This concept may be of importance when considering the administration of L-DOPA by intestinal gel infusion, which may act directly on GI tract [202].

## 4. Discussion

Despite increased interest in the recent years in PD-associated NMS, there is still a paucity of knowledge on the GI features of PD. This is an unfortunate state of affairs since these features are more difficult to manage than motor symptoms and are therefore of great concern for parkinsonian patients. In addition to their adverse effects on quality of life, GI problems are even more relevant to the understanding of the etiology of PD, insofar as Braak's hypothesis holds true. Accordingly, by collecting more clinical data on peripheral symptoms in putative cases of PD, an early diagnosis and better preventive action, as well as more efficient management of this disorder at its critical initiation and development stages, might be possible. For the time being, such a therapeutic approach is still purely speculative since PD is diagnosed solely following the recurrent manifestation of motor symptoms. Therefore, inasmuch as the importance of the ENS is further confirmed by future PD research, it might become essential to target the earliest manifestations of the disease in order to delay or even prevent neurodegeneration and thus the apparition of motor symptoms in PD patients.

This review summarizes the range of effective as well as potential therapeutic approaches to the management of GI symptoms in PD patients. Unfortunately, all existing treatments for both motor and nonmotor symptoms are purely symptomatic and result in merely temporary relief of these manifestations. Furthermore, it is very difficult to adequately treat GI symptoms because the exact target remains often unknown due to the lack of basic knowledge on the pathophysiology of the ENS component in the etiology of PD. Indeed, the main objective of current therapeutic research on PD is still oriented towards its management within the limits of present knowledge, that is, mainly reducing the side effects of medication, rather towards the further investigation of PD pathogenesis.

To date, several hypotheses have been proposed to understand the GI aspects in the physiopathology of PD. The most promising among these hypotheses include neurodegeneration, *α*-syn overexpression, inflammation, intestinal hyperpermeability, and microbiota disturbance as likely mechanisms involved in GI dysfunction [83, 84, 99, 204–208]. Furthermore, some factors have been suggested to participate in the initiation of the PD process, namely, disruption of the lysosomal and proteasomal systems, abnormal autophagy, endoplasmic reticulum stress, mitochondrial dysfunction, and oxidative stress [209–215]. Unfortunately, none of the latter putative factors could be confirmed as a PD biomarker due to the lack of an animal or cellular model that faithfully reproduces all features of PD. In the current state of our basic knowledge on PD pathophysiology, more optimal therapeutic avenues might be obtained by targeting a subset of these elements, given the fact that PD is clearly a multifactorial disease. However, a better insight into the etiology and mechanisms of the disease is crucial in order to find more targeted and effective treatments.

As summarized in the present review, there are now several lines of evidence that clearly demonstrate that GI dysfunctions not only are painful symptoms whose treatment constantly challenges clinicians, but also are relevant to the very process that causes PD, likely as reflections of processes that are under control by the ENS. Thus, GI symptoms in PD definitely should deserve much closer attention and warrant more detailed investigation in order to grasp the causative mechanisms at the core of this complex disease, which is a necessary prelude to the proper management of the disease's symptoms and, ultimately, to an actual curative strategy. Undoubtedly, further critical aspects of the mechanism leading to PD remain to be discovered and should call for a reassessment of the whole medical approach to this devastating disorder. Thus, in view of the recent developments in PD research emphasized in the present coverage of the literature, the peripheral aspects of PD should remain a priority in order to improve the therapeutic approaches to the disease, which are clearly in need of major improvements.

## Figures and Tables

**Table 1:** GI physiopathologic manifestations in PD. Summary of clinical studies exploring Lewy bodies expression and/or presence of neurodegeneration in enteric nervous system of parkinsonian patients.

Studies	GI part	Plexi	Disease stage or duration	Symptoms (number of PD patients)	GI pathological observations (number of PD patients)
Qualman et al., 1984 [80]	Esophagus and colon	Myenteric	Unknown		Lewy bodies (2/3)

Kupsky et al., 1987 [81]	Colon	Myenteric	Unknown	Megacolon (1/1)	Lewy bodies (1/1)

Wakabayashi et al., 1988 [82]	Upper esophagus to the rectum	Myenteric and submucosal	From less than 1 year to 27 years		Intraneuritic Lewy bodies in myenteric neurons of the esophagus (7/7), stomach (2/7), duodenum (2/7), jejunum (1/7), colon (1/7), and rectum (1/7) Intraneuritic Lewy bodies in submucosal neurons of the jejunum (1/7), colon (2/7), and rectum (1/7) Intracytoplasmic Lewy bodies in myenteric neurons of the esophagus (1/7)

Wakabayashi et al., 1990 [83]	Upper esophagus to the rectum	Myenteric and submucosal	8 years, 27 years, and unknown		Almost all neurons containing Lewy bodies were TH^+^or VIP^+^ (3/3) No apparent loss of TH^+^ and VIP^+^ neurons cell bodies and process

Singaram et al., 1995 [99]	Ascending colon	Myenteric and submucosal	Longstanding severe disease (>20 years for 8 patients)	Megacolon (9/11) Colon cancer (1/11) Needed manual evacuation (7/11)	Decrease in DAergic neurons number (9/11) Lewy bodies in myenteric neurons (11/11) ^*∗*^ *Mostly observed in VIP and TH* ^*+*^ * neurons* Decrease in DA concentration No difference in TH^+^, VIP^+^, and total neurons number

Braak et al., 2006 [84]	Distal esophagus and stomach	Myenteric and submucosal	Stage 2 to stage 5		Intraneuronal Lewy bodies (5/5)

Lebouvier et al., 2008 [102]	Ascending colon	Submucosal	>5 years	Constipation	Lewy neurites (4/5) No difference in TH^+^ and total neurons number

Beach et al., 2010 [79]	Upper esophagus to the rectum, submandibular gland, liver, pancreas, and gallbladder	Myenteric and submucosal	More than 80% in stage 3 or 4		Lewy bodies inclusions (11/17) ^*∗*^ *14/15 patients for only esophagus and submandibular gland*

Lebouvier et al., 2010 [89]	Ascending and descending colon	Submucosal	Group 1: ≤6 years (9 patients) Group 2: 7–12 years (10 patients) Group 3: ≥13 years (10 patients)	Chronic constipation ^*∗*^ *More frequent among patients with Lewy neurites*	Lewy neurites (21/29) ^*∗*^ *Group 1 = 7; Group 2 = 5; Group 3 = 9* ^*∗*^ *Proportion of patients with Lewy pathology did not correlate with disease progression but positively correlated with age* ^*∗*^ *60% were found in TH* ^*+*^ * neurons* Decrease in total neurons number

Annerino et al., 2012 [92]	Stomach, duodenum, ileum, transverse colon, and rectum	Myenteric	From 4 to 22 years		Lewy bodies (12/13) Lewy neurites (13/13) ^*∗*^<*3% were found in TH* ^*+*^ * neurons* ^*∗*^ *No correlation with age or disease progression* No difference in TH^+^, VIP^+^, NOS^+^, and total neurons number

Pouclet et al., 2012 [90]	Ascending and descending colon and rectum	Submucosal	From 1 to 24 years		Lewy neurites in ascending colon (17/26), in descending colon (11/26), and in rectum (6/26)

Pouclet et al., 2012 [91]	Descending colon	Submucosal	From 3 to 15 years		Lewy neurites (4/9)

Shannon et al., 2012 [86]	Sigmoid colon	Submucosal	From 6 months to 8 years	Mild disabilities	*α*-syn positive staining (9/9)

Gold et al., 2013 [87]	Colon	Myenteric and submucosal	Unknown		*α*-syn positive staining (10/10) ^*∗*^ *Higher prevalence and grade of α-syn detectability than controls*

Hilton et al., 2014 [85]	Esophagus, stomach, small intestine, colon, and gall bladder	Submucosal	From 8 years prior to the onset of motor symptoms to 15 years after diagnosis	Postural hypotension, constipation, dysphagia, urinary incontinence, impotence, nocturia, and drooling	*α*-syn positive staining (7/62) ^*∗*^ *11% in “postdiagnosis” tissues, 7% in “up to 5 years prior to diagnosis” tissues, 17% in “5–10 years prior to diagnosis” tissues, and 0% in “more than 10 years before diagnosis” tissues* ^*∗*^ *Proportions of positive biopsies in both the upper and the lower GI tract were similar*

Gelpi et al., 2014 [93]	Distal esophagus, stomach, ileum, colon, and rectum	Myenteric	Average of 11.5 years ^*∗*^ *Average of 18 years for PD patients with dementia*	Dementia (6/10)	Lewy neurites and Lewy bodies inclusions in distal esophagus, stomach, and colon (8/10)

Corbillé et al., 2014 [103]	Ascending and descending colon	Submucosal	From 1 to 24 years		No difference in TH^+^ and total neurons number

Beach et al., 2016 [96]	Sigmoid colon	Myenteric and submucosal	Average of 15.2 years		*α*-syn positive staining in the submucosal (5/5) and myenteric (4/5) plexi

^*∗*^: note.

**Table 2:** Effective therapeutic approaches. Classification and mechanisms of action of the various effective options for treating GI symptoms experienced by PD patients, depending on efficacy and side effects.

GI symptoms	Classification	Therapeutic approaches	Mechanisms of action	Dosage (adult)	Efficacy (on patients)	Side effects (% of patients)	Comments	Studies
*Constipation*	*(1) Use with caution*	Tricyclic antidepressants	Anticholinergic side effects					[15, 52]
		Antimuscarinics	Anticholinergic side effects					[15, 52]
		Opioids	Anticholinergic side effects					[15, 52]
	*(2) Nonpharmacological options*	Exercise	Intestinal stimulation by movements, increased fluids, and muscular mass					[6, 16, 32]
		Dietary fibers						[6, 16, 32]
		Increased fluid uptake						[6, 16, 32]
	*(3) Laxatives*	Macrogol (polyethylene glycol)	Passes through the gut without being absorbed and digested by enzymes, causing retention of water in the intestinal tube	Oral: 17 g (~1 tablespoon) dissolved in 240 mL of water or juice* once daily*		Abdominal bloating, cramping, diarrhea, flatulence, and nausea	Do not use for >1-2 weeks	[121, 189]
		Lactulose	Passes through the gut without being absorbed and digested by enzymes, causing retention of water in the intestinal tube	Oral or rectal: 10 to 20 g, *daily*		Abdominal discomfort and distention, belching, cramping, diarrhea (excessive dose), flatulence, nausea, and vomiting		[190]
		Magnesium sulfate	Blocks peripheral muscular contractions and neurotransmission	Oral: 2–4 level teaspoons of granules dissolved in 240 mL of water; *may repeat in 6 hours*		Hypermagnesemia, flushing, hypotension, and vasodilatation	Do not exceed 2 doses per day	[191]
		Bisacodyl	Stimulates enteric nerves to cause colonic contractions	Oral or rectal: 5–15 mg as *single dose*		<*1%*: abdominal mild cramps, metabolic acidosis or alkalosis, hypocalcemia, nausea, rectal irritation, vertigo, and vomiting		[124]
		Sodium picosulfate	Stimulates peristalsis and promotes water and electrolytes accumulation in the colon	Oral: 150 mL in the evening before the colonoscopy, followed by a second dose ~5 hours before the procedure		Hypermagnesemia (*12%*), hypokalemia (*7%*), increased serum creatinine (*5%*), hypochloremia (*4%*), hyponatremia (*4%*), headache (*3%*), nausea (*3%*), and vomiting (*1%*)	Mainly used for colonoscopy procedure	[124]
		Docusate sodium (alone or in combination with psyllium)	Unclear; may inhibit fluids absorption or stimulate secretion in jejunum	Oral: 50 to 360 mg, *once daily* or in divided doses		Throat irritation (*1 to 10%*)		[192]
		*Senna acutifolia*	Reduces fluid absorption from the faeces and influences fluid secretions by the colon				Long-term use is not recommended	[126]
	*(4) Other pharmacological options*	Lubiprostone	Intestinal ClC-2 chloride channel activator	Oral: 24 *µ*g *twice daily*	*64%*	Intermittent loose stools (*48%*), nausea (*29%*), diarrhea (*12%*), abdominal pain (*8%*), flatulence (*6%*), dizziness (*3%*), and vomiting (*3%*)		[126, 127]
		Methylnaltrexone	*µ*-Opioid antagonist	Subcutaneous: 12 mg, *once daily*	*60%*	Abdominal pain (*45%*), flatulence (*33%*), diarrhea (*30%*), and nausea (*24%*)	Discontinue all laxatives prior to use; if response is not optimal after 3 days, laxative therapy may be reinitiated	[128]
		Linaclotide	Guanylate cyclase C agonist	Oral: 145 *µ*g, *once daily*		Abdominal cramping (*4%*), discomfort (*4%*), and diarrhea (*4%*)	Contraindicated in pediatric patients (<6 years of age)	[129, 130, 193]

*Drooling and dysphagia*	*(1) Use with caution*	Cholinesterase inhibitors						[51]
		Clozapine	Serotonin antagonist				Demonstrated effectiveness against dyskinesias	[51, 161, 194]
		Yohimbine	Presynaptic *α*2-adrenergic blocking agent					[51, 162]
		Quetiapine	D2 receptors (mesolimbic pathway) and 5HT2A (frontal cortex) antagonist				Demonstrated effectiveness against dyskinesias	[51, 195]
	*(2) Nonpharmacological options*	Chewing gum or sucking on hard candy			*5 times improved*			[158]
		Speech and position therapy					Self-motivation is an important factor to obtain a positive outcome	[159, 160]
	*(3) Pharmacological options*	Botulinum toxin A/B injections (parotid and submandibular glands)	Inhibits the cholinergic parasympathetic and postganglionic sympathetic activity	*A toxin:* 500 units divided among affected glands		*A toxin:* dryness of mouth and mild transitory swallowing difficulties (*6%*)	Produced by *Clostridium botulinum* bacterium	[163, 165, 166, 168]
				*B toxin:* 1,000 units into each parotid gland and 250 units into each submandibular gland		*B toxin:* dryness of mouth (*40%*), worsened gait (*25%*), diarrhea (*15*%), neck pain (*15%*), and mild transitory swallowing difficulties (*16%*)		[50, 163, 167]
		Atropine ophthalmic drops (sublingual administration)	Anticholinergic that blocks muscarinic receptor M3	1 drop of 1% atropine solution, *twice daily for 1 week*		Hallucinations (*29*%) and delirium (*14%*)	Lack of clinical evidence for treatments lasting longer than a few weeks Use with caution in the elderly; increased risk for anticholinergic effects, confusion, and hallucinations	[170]
		Glycopyrrolate	Anticholinergic that blocks muscarinic receptor M3	Oral: 1 mg 3 times, *daily*	*95 to 100%*	Dry mouth (*52%*), urinary retention (*13%*), vision problems (*13%*), constipation (*13%*), and nausea (*4%*)		[171, 172, 174, 175]

*Nausea, vomiting and gastroparesis*	*(1) Use with caution*	High-fat foods						[31]
		Metoclopramide	Dopamine antagonist				Contraindicated for PD patients because it worsens motor symptoms by blocking dopamine receptors in the CNS	[31]
	*(2) Nonpharmacological options*	Small and frequent meals						[31]
		Drinking during meals						[31]
		Walking after meals						[31]
	*(3) Pharmacological options*	Domperidone	Dopamine antagonist	Oral: initiating at 10 mg 3 times, *daily* (maximum: 30 mg/day)	*100%*	Xerostomia (*2%*) and headache (*1%*)	Does not readily cross the BBB ^*∗*^ *Use the lowest effective dose for the shortest duration necessary* ^*∗*^ *Not available in the United States*	[149, 196, 197]
		Trimethobenzamide	Unclear; most likely involves the chemoreceptor trigger zone (through which emetic impulses are transported to the vomiting center)	Oral: 300 mg; intramuscular: 200 mg, *3 or 4 times daily*	*20%*	Dizziness, headache, blurred vision, and diarrhea	May mask toxicity of other drugs or conditions	[198]

^*∗*^: note.

## Abbreviations

*α*-syn: Alpha-synuclein
BBB: Blood-brain barrier
BTX: Botulinum toxin
CNS: Central nervous system
DA: Dopamine
DAergic: Dopaminergic
DBS: Deep brain stimulation
ENS: Enteric nervous system
GI: Gastrointestinal
L-DOPA: Levodopa, L-3,4-dihydroxyphenylalanine
NMS: Nonmotor symptoms
NOS: Nitric oxide synthase
PD: Parkinson's disease
UPDRS: Unified Parkinson's Disease Rating Scale
VIP: Vasoactive intestinal peptide.
